# Advanced Stainless Steel—From Making, Shaping, Treating to Products

**DOI:** 10.3390/ma18204730

**Published:** 2025-10-15

**Authors:** Chao Chen, Zhixuan Xue, Wangzhong Mu

**Affiliations:** 1 College of Materials Science and Engineering, Taiyuan University of Technology, Taiyuan 030024, China; 2024510282@link.tyut.edu.cn; 2 Key Laboratory of Electromagnetic Processing of Materials (Ministry of Education), School of Metallurgy, Northeastern University, Shenyang 110819, China; 3 Department of Materials Science and Engineering, KTH Royal Institute of Technology, Brinellvägen 23, SE-100 44 Stockholm, Sweden

**Keywords:** stainless steel, steelmaking, inclusions, continuous casting, heat treatment, corrosion, fatigue

## Abstract

Stainless steels have undergone more than a century of continuous development, during which various advanced grades—such as lean duplex, super austenitic, and high-nitrogen stainless steels—have been introduced. Despite remarkable progress, the manufacturing of stainless steel remains a complex process that spans multiple critical stages, including stainless steelmaking, solidification and casting, continuous casting, heat treatment, electroslag and vacuum arc remelting, as well as both hot and cold rolling operations. Ensuring excellent corrosion resistance and mechanical performance of the final products continues to be a central focus of research and production. The current Special Issue (SI) entitled ‘*Advanced Stainless Steel—from Making, Shaping, Treating to Products*’ has collected eight research papers focusing on various aspects of steel production, e.g., inclusions in steelmaking and continuous casting processes, continuous casting processes and the quality of stainless steel casting, heat treatment, corrosion of steels, and fatigue of steels. This summary aims to contribute to the state-of-the-art of the development of steel production.

## 1. Introduction

In the 1820s, scientists Pierre Berthier, Stoddard, and Faraday noted that chromium-containing iron was more corrosion-resistant against certain acids than its non-chromium counterpart [1]. Their alloys, however, were not true stainless steel. This era is widely attributed to the Englishman Harry Brearley, who in 1913 developed and analyzed the first authentic stainless steel, with a composition of 12.8% chromium and 0.24% carbon [1].

Owing to their superior combination of corrosion resistance and mechanical strength, stainless steels are extensively applied in engineering fields such as petrochemical industries, machines, railway systems, and aerospace applications. In modern classifications, stainless steels are generally divided into austenitic, ferritic, martensitic, and duplex categories [2,3]. Moreover, continuous alloy design and processing optimization have led to the emergence of new grades, including lean duplex, super austenitic, and high-nitrogen variants, which further expand the performance envelope of stainless steels. There are some recent book chapters [2], monographs [3], and review papers [4,5,6,7,8] that summarize the recent developments in stainless steels.

To name a few of the novel applications of stainless steels, the super austenitic stainless steel has been a hot research topic [9,10,11,12,13,14,15,16] nowadays due to its difficulty in continuous casting and precipitation controls. In addition, the 316 group stainless steel is tailored and applied in the in-core and out-of-core components of Generation-IV fast reactors [17,18,19], nuclear power pipeline systems [20,21], and low magnetic scenes [22,23], for example, medical materials [24,25] and mobile phone frames [26,27,28]. The stainless steel foils are another application in the communication industry [29]. Recent composite materials are made by ultra-thin stainless steel foils and carbon fiber reinforced polymer (CFRP) [30,31]. These advanced lightweight materials are promising for aircraft. In addition, composite plates made by stainless steel and titanium alloy or other steel grades are studied and produced in many areas [32,33,34]. A novel potential functional application of stainless steels is the bifunctional water electrolysis electrode [35,36], which is under development.

The current production process of stainless steel is basically as follows [37,38]: blast furnace→hot metal pretreatment→converter steelmaking→ladle furnace and/or vacuum oxygen degassing→tundish→continuous casting→hot rolling→pickling and annealing→cold rolling. Alternatively, the electric arc furnace (EAF) process can replace the steps before converter steelmaking [39]. In addition, some special melting methods, such as electric slag remelting and vacuum arc remelting, are used in the production of special stainless steels [21].

For the Argon Oxygen Decarburization (AOD) [40,41,42] and Vacuum Oxygen Degassing (VOD) [43] furnace operations, the technologies are very mature. The special design of the tundish for stainless steel is summarized in ref [44,45]. The design of the submerged entry nozzle (SEN) for stainless steel production is still an ongoing work [46]. Working roll modification is another hot research topic for rolling stainless steels [47,48].

The production of stainless steel is still a challenging process with respect to the non-metallic inclusions [49,50,51,52,53,54] and casting defects [55,56,57]. The inclusions formation thermodynamics [58], as well as nozzle clogging during the production of various stainless steel grades, for example, high silicon [59,60], Ti stabilized [61,62], and rare earth metal treated [63,64,65], are problems to be solved. The relationship between corrosion and inclusions in stainless steel is studied [66,67]. Solidification structure refining [68] for ferritic stainless steels, residual ferrite in austenitic stainless steels [69], and phase precipitation in duplex and super austenitic stainless steels [26,70,71,72] are the issues with respect to casting defects. The heat treatment of stainless steels [73,74,75] as well as the mechanical properties, corrosion resistance [76,77,78,79,80] of the product are all important issues.

## 2. An Overview of Published Articles

Manuscripts on many subjects regarding (stainless) steel production were submitted for the current Special Issue (SI). After the peer-review process, eight papers were finally accepted for publication. The papers cover the topics of inclusions in steelmaking and continuous casting processes, continuous casting processes and the quality of stainless steel casting, heat treatment, corrosion of steels, and fatigue of steels. A detailed description of each contribution is provided in Table 1.

Contribution 1 [81] established a three-dimensional segmented coupling model to analyze continuous casting billets under the simultaneous influence of final and mold electromagnetic stirring (F-EMS and M-EMS). Model accuracy was verified by comparing carbon segregation behavior in billets with and without electromagnetic stirring through industrial trials. Results demonstrated that both F-EMS and M-EMS generate tangential molten steel flow, which modifies the solidification dynamics and solute distribution inside the billet.

Contribution 2 [82] investigated the precipitation behavior of Ce inclusions in steel melt and predicted their evolution using thermodynamic calculations. It was found that varying Ce levels alter the precipitation sequence of rare earth inclusions, where the formation of CeO_2_, Ce_2_O_3_, and CeAlO_3_ is increasingly suppressed with higher Ce contents. The addition of Ce also refined the grain size and pearlite lamellae of U75V steel. Mechanical testing indicated that when Ce content is controlled below 0.0042%, the tensile strength and impact toughness of U75V steel are both enhanced.

Contribution 3 [83] analyzed the low-cycle fatigue behavior of S420M steel under different loading states, including undeformed and pre-strained specimens. Under strain-controlled fatigue, S420M steel exhibited no cyclic stabilization phase. After the initial deformation, significant variations in cyclic behavior were observed from hysteresis loop parameters. The fatigue life of pre-strained samples differed from that of unstrained ones, showing dependence on the applied loading conditions.

Contribution 4 [84] focused on inclusion characteristics in high-titanium steel casting billets (0.4% Ti, 0.004% N) by means of industrial sampling and comparative cross-sectional analysis at central and quarter thickness positions. Results showed that accelerating cooling rates and lowering titanium levels effectively delayed TiN precipitation, thereby suppressing the formation of coarse TiN inclusions in high Ti steels.

Contribution 5 [85] explored the distribution and evolution of residual ferrite in 304L austenitic stainless steel continuous casting slabs. Along the thickness direction at the slab’s width center, ferrite content exhibited an “M”-type distribution—approximately 3% near edges and up to 13% at the center. Thermodynamic evaluation indicated an FA solidification mode. The impact of heat treatment processes on the ferrite content was also investigated. Optimal heat treatment at 1250 °C for 48 min followed by air cooling minimized ferrite content while maintaining the characteristic “M”-shaped distribution.

Contribution 6 [86] applied statistical modeling methods, including linear regression and artificial neural networks (ANNs), to predict the corrosion response of austenitic stainless steels (316L, 904L, and AL-6XN) under varying environmental factors. The considered parameters included temperature (30–90 °C), chloride ion concentration (20–40 g/L), and pH (2–6). The detailed analysis of variance (ANOVA) confirmed that the Pitting Resistance Equivalent Number (PREN, 24–45) exerted the most significant influence on the critical pitting potential, followed sequentially by temperature, pH, and chloride concentration.

Contribution 7 [87] explored the formation of akaganeite during atmospheric corrosion induced by NaCl deliquescence through laboratory simulations. The deliquescence of NaCl particles produced local electrolyte droplets, within which corrosion occurred independently. Akaganeite was detected within 12 h of exposure, while lepidocrocite and magnetite, as early corrosion products, facilitated its formation. Moreover, the extent of salt deposition was identified as a crucial factor influencing macroscopic akaganeite accumulation.

Contribution 8 [88] performed multi-factor coupled constant-amplitude fatigue tests on Q500qENH weathering steel V-groove welded joints and established an equivalent finite element model to analyze interdependent parameters under coupled conditions. The results demonstrated that stress level had the most pronounced impact on fatigue behavior, followed by corrosion duration and ambient temperature. Simulation findings further indicated that low temperatures improved the fatigue life of slightly corroded specimens by approximately 20%, though progressive damage occurred before reaching peak service performance.

## 3. Summary

The current Special Issue (SI), *Advanced Stainless Steel—from Making, Shaping, Treating to Products*, collects the research contributions on the topics of inclusions in steelmaking and continuous casting processes, continuous casting processes optimization and the quality of stainless steel castings, heat treatment, corrosion of steels, and fatigue of steels. Both experimental, numerical simulation, and artificial neural network model studies on the stainless steel production topics were reported in different papers. As a pity, review papers on this topic are not presented in this SI. We may organize a second Volume II SI with the identical topic to invite and collect more contributions regarding different topics of stainless steel production.

## Figures and Tables

**Table 1:** Summary of the published contributions in this Special Issue.

No. of Contribution	Research Area	Focus	Type of Research
1 [81]	Continuous casting	Model comparison on the performance of combined mold and final electromagnetic stirring (M-EMS, F-EMS)	Numerical model study
2 [82]	Inclusions modification	Effect of the addition of Ce in U75V steel on the inclusion precipitation and mechanical properties	Experimental study
3 [83]	Fatigue of steel specimen	The low-cycle fatigue of S420M steel under undeformed and pre-strained conditions	Experimental study
4 [84]	Inclusions in continuous casting slab	A full section comparative analysis of the inclusions in a high-titanium steel	Experimental study
5 [85]	Continuous casting and heat treatment of stainless steel	Residual ferrite distribution in 304L austenitic stainless steel slab and the effect of heat treatment on it	Experimental study
6 [86]	Corrosion of stainless steel	Predict the corrosion behavior of austenitic stainless steels (316L, 904L, and AL-6XN) under various environmental conditions	Experimental study and artificial neural network models
7 [87]	Corrosion of steel	Formation of akaganeite in atmospheric corrosion induced by NaCl deliquescence	Experimental study
8 [88]	Fatigue of steel	Fatigue tests on Q500qENH weathering steel V-groove welded joints and finite element model study	Experimental study and numerical model study
